# Effect of microbial interaction on flavor quality in Chinese baijiu fermentation

**DOI:** 10.3389/fnut.2022.960712

**Published:** 2022-08-03

**Authors:** Lei Gao, Jian Zhou, Guiqiang He

**Affiliations:** ^1^School of Life Science and Engineering, Southwest University of Science and Technology, Mianyang, China; ^2^Engineering Research Center of Biomass Materials, Ministry of Education, Southwest University of Science and Technology, Mianyang, China

**Keywords:** Chinese baijiu, flavor quality, microbial interaction, synergistic fermentation, synthetic microbiota

## Abstract

Chinese baijiu brewing is an open, complex, and synergetic functional microbiota fermentation process. Microbial interaction is pivotal for the regulation of microbial structure and function in the brewing microecosystem, consequently affecting the flavor and quality of baijiu. This article mainly summarizes the effect of microbial interactions among functional microbiota on the growth performance, flavor formation, and safe quality of baijiu fermentation process. In addition, the review specifically emphasizes on the microbial interactions for the regulation of “Ethyl Caproate-Increasing and Ethyl Lactate-Decreasing” in Chinese strong-flavor baijiu. Furthermore, the construction of synthetic microbiota by metabolic characteristics of the functional microbes and their interactions for regulating and controlling flavor quality of Chinese baijiu is also reviewed and prospected.

## Introduction

Chinese baijiu, one of the well-known traditional fermented foods, possesses strong ethnic characteristics in Chinese culture and industrial advantages in the national economy. For example, owing to its unique flavor and aroma, the output and revenue of Chinese baijiu achieved 7.1 billion liters and 583.6 billion RMB in 2020, respectively, reaching a total profit of 131.2 billion RMB. The distinctive flavor and taste of Chinese baijiu is attributed to the composition and proportion of multifarious flavor compounds. Typically, four organic acids (acetic acid, lactic acid, butanoic acid, and hexanoic acid) and their corresponding ethyl esters, especially, caproic acid and ethyl caproate, have been confirmed to be dominant compounds and important contributors to the characteristic flavor of strong-flavor baijiu (1, 2). In fact, 12 flavor types of Chinese baijiu contain more than 1,870 flavor compounds, namely, acids, alcohols, esters, ketones, aldehydes, aromatics, nitrogenous compounds, terpenes, and sulfur compounds (3). In addition, the potential functional component in Chinese baijiu was also uncovered in recent years. For instance, a tetrapeptide (Ala-Lys-Arg-Ala) had been successfully identified in sesame-flavor baijiu and exhibited preventive effects against 2,2-Azobis (2-methyl-propanimidamidine) dihydrochloride-induced oxidative stress in HepG2 cells (4).

The formation of these flavor and functional compounds is extremely complicated and can be fluctuated mainly by dynamic succession of functional microbiota during the fermentation process. These functional microbes are supposed responsible for the production of flavor compounds by their extensive interactions. For example, the flavor compounds, namely, fatty acids, esters, terpenes, and aromatic compounds produced by *Saccharomyces cerevisiae* were correlated with the mixing ratio of *Bacillus licheniformis* in Chinese maotai−flavor baijiu fermentation (5). Moreover, the microbial interaction is a crucial factor for maintaining the co-occurring in microbiota structure, which will influence the microbial metabolism and flavor formation during the wine fermentation (6). Therefore, revealing the mechanism of the microbial interactions on flavor metabolism is important for regulation of Chinese baijiu fermentation. Based on this, some related studies have already been focused on the microbial interactions and how to achieve the targeted regulation by these interactions in baijiu fermentation (7, 8).

**FIGURE 1:**
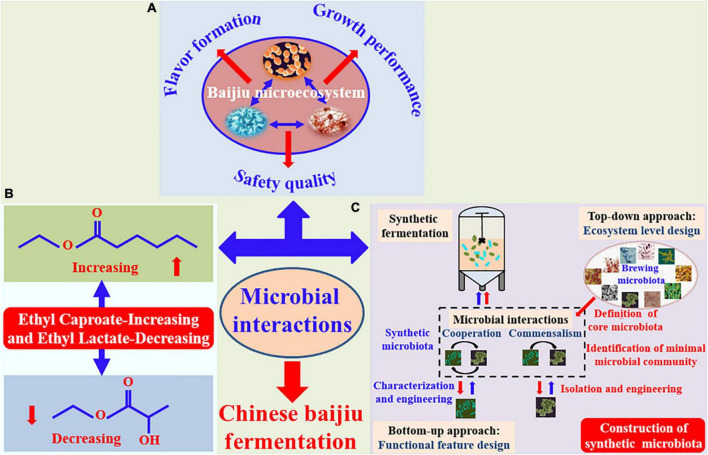
Effect of microbial interactions on regulation of growth and flavor metabolism **(A)** and “Ethyl Caproate-Increasing and Ethyl Lactate-Decreasing” **(B)**, as well as construction of synthetic microbiota (“top-down” approach was red arrows, and “bottom-up” approach was blue arrows) **(C)** in Chinese baijiu fermentation process.

In this review, recent researches relating to the effect of microbial interactions on growth metabolism, flavor formation, and safe quality in Chinese baijiu fermentation (Figure 1A), especially for regulation of “Ethyl Caproate-Increasing and Ethyl Lactate-Decreasing” in strong-flavor baijiu (Figure 1B) are summarized and discussed. Furthermore, the construction of synthetic microbiota by considering the metabolic features of functional microbes and their interactions (Figure 1C), is also described and prospected in the regulation of flavor quality for Chinese baijiu fermentation.

## Interactions among functional microbes

Chinese baijiu is produced by the traditional spontaneous solid-state fermentation process containing various functional microbes and their complex interactions (9). In general, microbial interactions are mainly classified through ecological typing into competition, mutualism (cooperation), commensalism, amensalism, or parasitism, and these interactions can be regulated by modifying the metabolic pathway, intercellular communication, and spatial structures, thereby accomplishing the specific functions (10). In fact, there are mainly synergistic (cooperation) and antagonistic (competition) effects involved in the microbial interactions in the baijiu brewing microecosystem. Here, we describe the effect of interactions among functional microbes on the growth performance, flavor formation, and safety characteristic (Figure 1A) in the baijiu fermentation process.

### Effect of microbial interactions on growth performance

In fact, microbial interactions are usually deemed as cooperative networks with functional microbiota working together toward an ultimate goal during the baijiu-brewing process. This implicates that the cooperation and interaction can influence growth performance and even metabolic activity of the microbial consortia. For example, the biomass of *S. cerevisiae* increased when it was co-cultured with *Aspergillus oryzae* at the ratios of 1:0.1, 1:0.5, and 1:1, and this was attributed to providing more glucose for *S. cerevisiae* growth by inducing enzyme system of starch hydrolyzation in *A. oryzae* (11). This means that the metabolites produced by microbe have synergistic or antagonistic effect on other microbes, also known as metabolite regulation mechanism of the microbial interactions. But, on the contrary, the antagonistic interaction between both species was also uncovered. The growth and biomass of *S. cerevisiae* and *A. oryzae* were inhibited in the mixed culture system, but the protein synthesis for the cell wall of *S. cerevisiae* was significantly promoted (12). In addition, the occurrence and effectiveness of synergistic interactions within functional microbiota are affected easily and restricted by the environmental factors in the natural succession process (13). For instance, the growth of *Zygosaccharomyces bailii* was normal at 30°C, but was inhibited at 37°C in the co-culture system with *B. licheniformis* for Chinese maotai-flavor baijiu fermentation (14). This is mainly related to the stress mechanism of fermentation environment, that is, the growth and survival of brewing microbiota are declined under multiple environmental stresses, namely, alcoholic, acidic, thermal, and oxidative during baijiu production.

Most notably, the antagonistic effect between non-*Saccharomyces* yeasts and *S. cerevisiae* is essential for Chinese baijiu brewing. Many researches have demonstrated that *S. cerevisiae* could inhibit the growth of non-*Saccharomyces* such as *Z. bailii* (15), *Wickerhamomyces anomalus* (16), and *Issatchenkia orientalis* (17) when they were co-cultured. These interactions might be resulted from the non-specific competition for nutrients among yeasts (18) and the inhibition of metabolites (such as ethanol) produced by *S. cerevisiae* (19). So, microbial interactions inevitably influence the growth performance of brewing microbiome during the baijiu fermentation process, consequently altering the metabolic activity and even the flavor formation of the final products.

### Effect of microbial interactions on flavor formation

There are multifarious strategies for improving the flavor quality of traditional fermented foods (20, 21), and the core point of strategy is regulation of the microbial community and their interactions in the fermentation process. For example, the contents of caproic acid and ethyl caproate were improved in strong-flavor baijiu microecosystem by increasing the abundances of caproic acid bacteria and methanogens and also the hydrogen transfer interaction among them with fortified Daqu fermentation (22). Interestingly, although the growth performances of functional strains are repressed, their activities of flavor metabolism are not weakened in the co-culture fermentation system. For instance, when *Z. bailii* and *B. licheniformis* were coexisted, the growth of *B. licheniformis* was significantly inhibited, but the genes, namely, *GAPDH*, *PGM1*, *ENO1*, *PDC1*, *COX1*, and *MEP2* involved in glycolysis, Ehrlich, and oxidative phosphorylation pathways in *Z. bailii* were upregulated, thereby producing more alcohols, acids, esters, and aldehydes in co-culture (15). Actually, the inhibition of growth but promotion of flavor metabolism activity in the co-culture system with non-*Saccharomyces* yeasts and *S. cerevisiae* is ubiquitous in the baijiu fermentation process (15–17). For instance, compared with the single culture of *W. anomalus*, higher yield of ethyl acetate was observed when *S. cerevisiae* and *W. anomalus* were co-cultured (16). This result indicated that the higher content of ethyl acetate could be attributed to synergy between non-*Saccharomyces* yeast and *S. cerevisiae* in co-culture (23). *S. cerevisiae* could produce acetic acid and ethanol, which were critical for *W. anomalus* in generating ethyl acetate in co-culture synergistic fermentation.

Besides aforementioned, some microbes have really poor ability to produce flavor compounds in the fermentation process, but they can coordinate the metabolic activity with those flavor producers. For example, *Bacillus amyloliquefaciens* and *Pichia membranaefaciens* were not effective flavor producers, but they could improve the flavor compounds with *S*. *cerevisiae*, *I. orientalis*, and *B. licheniformis* co-cultured in the sesame-flavor baijiu fermentation (17). In examples like this, the synergetic interactions between yeasts and lactic acid bacteria are the most widespread in the fermented alcoholic beverages (24). For instance, the content of ethyl lactate was significantly increased when co-cultured with *P. membranifaciens* and *Lactobacillus acetotolerans* in the strong-flavor baijiu fermentation compared with the mono-culture of *P. membranifaciens* (25). In addition, He et al. (26) reported that improvement of esters production and fruity flavor of strong-flavor baijiu was observed when fermented with the fortified Daqu in the brewing process, in which the synergistic interaction between *Lactobacillus* and *Candida* was considered to be an important driving factor. Another example of synergistic effect on higher production of 3-(methylthio)-1-propanol and dimethyl disulfide was also obtained by co-culturing *S. cerevisiae* with *Lactobacillus buchneri* in baijiu fermentation (27). This synergy mechanism between *S. cerevisiae* and *L. buchneri* was revealed by transcriptome analysis. *S. cerevisiae* upregulated expression of genes for generation of 3-(methylthio)-1-propanol and dimethyl disulfide in the presence of *L. buchneri*, which can regenerate the precursors methionine and S-adenosylmethionine by methyl recycle (27). On the contrary, yeast and lactic acid bacteria could provide nutrients to each other, and promote the growth metabolism of them (28).

### Effect of microbial interactions on safe quality

Although the traditional baijiu brewing has been produced for thousands of years, traditional hand-making in an open and complex brewing environment without strict control leads to low production, inconsistent quality, and even security risk (29). Based on the security of baijiu, current researches mainly pay attentions to the regulation and reduction of ethyl carbamate (EC), a class 2A carcinogen (30). For example, there are reports already that the concentrations of EC and its precursor cyanide effectively decreased in raw baijiu by pot still second distillation process (31, 32). Unfortunately, the flavor and quality of baijiu would be affected in part by altering the production process to eliminate the EC content. So, some researchers have focused on the microbial intervention methods for removing the EC, especially for microbial interactions (33, 34). For instance, low amounts of urea (the precursor of EC) was produced by *Lactobacillus* species with non-conventional yeasts, namely, *Pichia*, *Schizosaccharomyces*, and *Zygosaccharomyces* species co-fermentation in Chinese maotai-flavor baijiu (35). Moreover, Fang et al. (36) reported that EC generation by *S. cerevisiae* was significantly inhibited in the co-culture with *Lactobacillus brevis* and provided valuable insights into the molecular mechanism of EC formation by transcriptomic analysis.

Besides EC, some odor compounds were also detected in the baijiu fermentation process, thereby affecting the flavor and safety quality. For example, the compound *p*-cresol is the major off-odor and toxic component of strong-flavor baijiu (37). Another research indicated that *Clostridium* was the primary microbial source for *p*-cresol production and the formation of *p*-cresol could be inhibited by increasing the proportions of *Lactobacillus* (38). In particular, the concentration of *p*-cresol in sesame-flavored baijiu is decreased by hydrogen bond interactions with the non-volatile tetrapeptide Ala-Lys-Arg-Ala (39). Taken together, these studies inspire that intensification of interspecies interactions between *Clostridium* with relevant *Lactobacillus* or Ala-Lys-Arg-Ala-producing strains is a possible strategy for eliminating the *p*-cresol in baijiu fermentation.

## Regulation of “Ethyl Caproate-Increasing and Ethyl Lactate-Decreasing” by microbial interactions

At present, one of the tricky problems for many Chinese strong-flavor baijiu production enterprises is how to decrease lactic acid and increase caproic acid accumulation during the fermentation process (40). This technical challenge inevitably results in weakness of the body aroma by ethyl caproate, thinness of flavor and texture, and shortness of aftertaste, which consequently destroys the typical style of strong-flavor baijiu. To address this problem, many researches have been devoted to regulate and control the process parameters during the fermentation, such as scientific construction of fermentation cellar, optimization of pit mud, improvement the quality of fermentation starter, and regulation of the conditions for pit entry (41). However, these works focused mainly on uncovering the correlations between the lactic acid and caproic acid contents with the technical parameters in the baijiu fermentation process. The vital information related to the alteration of functional microbiota caused by these process parameters for regulation of “Ethyl Caproate-Increasing and Ethyl Lactate-Decreasing” is still fragmented.

According to this, some researchers have paid attentions on microbial interactions for regulation of “Ethyl Caproate-Increasing and Ethyl Lactate-Decreasing” (Figure 1B) in these years. For instance, the co-culture fermentation broth with *Clostridium kluyveri* and *Methanogen* was subjected to strong-flavor baijiu pit-entry fermentation, the concentration of caproic acid and ethyl caproate in the raw baijiu, respectively, increased by 114.7 and 142.8%, while that of ethyl lactate decreased by 64.1% (42). Interestingly, recent study indicated the relative abundance of caproic acid bacteria significantly increased after 15-day fed-batch fermentation with lactate as carbon sources, which meaned that the brewing microbiota exhibited a regular and directional evolutionary pattern for effectively achieving “Ethyl Caproate-Increasing and Ethyl Lactate-Decreasing” (43). However, in general, it is difficult to control the natural evolution of microbiota and easily fluctuated by environmental factors and process parameters.

Thus, it is of urgent need to develop feasible strategy for directionally regulating fermentation and accelerate the enrichment of functional microbiota. Most notably, an effective regulation approach is the interspecies hydrogen transfer interactions between the hexanoic acid producers and methanogenic archaea (44). The synergistic interaction between caproic acid bacteria and methanogens is extensively existed in baijiu-brewing microecosystem, which is conducive to maintain the stability of the microbial succession and also produce more caproic acid and ethyl caproate (42, 45, 46). In addition, considering the negative correlations between *Lactobacillus* and *Bacillus* reported by many researches, He et al. (22) performed a novel strategy for regulating strong-flavor baijiu fermentation by directional bioturbation with fortified Daqu (inoculation of *Bacillus subtilis* and *Bacillus velezensis*). The results demonstrated that the bioturbation by fortified Daqu was feasible for “Ethyl Caproate-Increasing and Ethyl Lactate-Decreasing” by interspecies interactions of functional microbiota, namely, *Bacillus*, *Lactobacillus*, *Caproiciproducens*, *Clostridium*, *Candida*, *Aspergillus*, *Methanobacterium*, and *Methanosarcina*.

## Construction of synthetic microbiota by the microbial interactions

Synthetic microbiota, constructed artificially by co-culturing of multiple species with well-defined genetic background and specific functions, is provided with low complexity, high controllability, and stability (47, 48). In recent years, some researchers have focused on how to transform to the synthetic fermentation from spontaneous fermentation for regulation of microbial metabolism and production of high-quality foods. For example, *Acetobacter pasteurianus*, *Lactobacillus brevis*, and *Lactobacillus fermentum* were co-cultured and constructed an acetoin-producing synthetic community, when it was applied in the traditional vinegar fermentation, the content of acetoin in vinegar increased significantly (49). In addition, it was reported that a tractable microbiota system was constructed by 24 widely distributed and culturable genera and conducted to the cheese rind fermentation (50). These studies provide references for synthetic microbiota in the different fermented foods. It means that the manipulation and repeatability of microbial succession and dynamic can be obtained *in vitro*. This also affords an opportunity to construct a synthetic microbiota system for food fermentation with prospective flavor and quality.

However, Chinese baijiu brewing is an open, complex, and synergetic of the functional microbiota fermentation process. It seems arduous to construct the synthetic microbiota for regulation and control of the flavor compounds formation in such a microecosystem. Fortunately, the methods of synthetic biology and microbiome make it possible by identification and isolation of the core microbiota with the development of modern biotechnology (51). For the construction of synthetic microbiota, revealing the phylogeny, metabolic functions, especially interspecies interaction of the selected strains are of paramount importance (52). Synthetic microbiota is generally constructed according to the top–down or bottom–up approach (Figure 1C), and the microbial interactions play an important role in the construction process by both the approaches (53).

For example, although the growth performances of *S. cerevisiae*, *P. membranaefaciens*, *I. orientalis*, *B. licheniformis*, and *B. amyloliquefaciens* were inhibited by each other, the co-culture of these five species could coordinate the metabolic activities by their interactions and produce the largest amount of flavor compounds in the Chinese sesame-flavor baijiu (17). For another example, the microbial interaction was analyzed by co-occurring network and the synthetic microbiota composed by *L. acetotolerans*, *Pichia kudriavzevii*, *Geotrichum candidum*, *Candida vini*, and *S. cerevisiae* was constructed, 77.27% of the flavor compounds produced by the synthetic microbiota exhibited a similar dynamic profile with that *in situ* system, and the flavor profile presented a similar composition (54). In recent, the core microbiota, namely, *Lactobacillus*, *Thermoactinomyces*, *Aquabacterium*, *Aspergillus*, and *Kazachstania* was identified in the fermented grains of Chinese strong-flavor baijiu by finding ubiquitous, dominant, flavor associated, and co-occurring microbiota together (55). And, the results lay the foundation of construction the synthetic microbiota for regulating the baijiu fermentation process and achieving the homogenization of product quality. Therefore, once we can understand the interaction mechanism of functional microbiota for constructing the synthetic microbiota, we will be able to regulate and control the fermentation process for production of high-quality baijiu.

## Conclusion

Chinese baijiu is produced by the spontaneous solid-state fermentation process involved in the multifarious microbes and their extensive and complex interactions. These interactions are propitious to flavor and safety improvement, such as “Ethyl Caproate-Increasing and Ethyl Lactate-Decreasing,” and to stability enhancement of baijiu-brewing microecosystem. Moreover, revealing the mechanism of microbial interactions is beneficial for rational construction of synthetic microbiota, and achieving the directional regulation for flavor quality in Chinese baijiu fermentation process.

## Author contributions

LG performed the literature search and wrote the manuscript. JZ performed the literature search and contributed to the manuscript revision. GH contributed to the literature summary, manuscript revision, and overall support of this work. All authors contributed to the article and approved the submitted version.
